# On the Dysmenorrhœa, Metrorrhagia, Ovaritis, and Sterility Associated with a Peculiar Form of the Cervix Uteri, and the Treatment by Division

**Published:** 1865-09

**Authors:** Robert Barnes


### ON THE DYSMENORRHŒA, METRORRHAGIA, OVARITIS, AND STERILITY ASSOCIATED WITH A PECULIAR FORM OF THE CERVIX UTERI, AND THE TREATMENT BY DIVISION.

By ROBERT BARNES, M. D.

The author described and figured the form of cervix uteri which projected into the vagina as a conical body, the vagina appearing to be reflected off at a point nearer the os internum than normal. The os externum was usually minute, scarcely admitting the uterine sound. This (the os externum) was the real seat of constriction. The os internum nominally was a narrow opening; and in these cases of dysmenorrhœa and sterility it was commonly found to be of normal calibre. It was, therefore, unnecessary to divide it, on account of the close proximity of the large vessels and plexuses running into the uterus on a level with it. The author maintained that this form of cervix was a cause also of retro- and peri-uterine hæmatocele, and of peritonitis. All these consequences might arise in single women. In the married state the evils enumerated were aggravated, and new ones arose. Women with this peculiarity were generally sterile; and if they became pregnant it was early in life, before the further consequences were developed. These were flexions, deviations, inflammation of the cervix and body, hypertrophy. Discussing the question of treatment, the author showed that dilatation was unsatisfactory; that incision of the os internum, as practised by Dr. Simpson's single bistourie caché, and by Dr. Greenhalgh's double bistourie caché, was unsafe and superfluous. He objected to the latter instrument, especially, that it must cut as it was set—that it was too much of an automatic machine, not leaving scope for the judgment of the operator. His (Dr. Barnes's) own instrument, constructed like a pair of scissors, acted on the same principle as Dr. Sims's; it divided only the os externum, so as to open the cavity of the cervix; the part to be cut being first seized between the two blades, the operation was perfectly free from risk; the hemorrhage was usually slight, and a good os was made. He had performed the operation many times, both in hospital and private practice, and was well satisfied with the results. One advantage of incision over dilatation was, that it relieved the engorgement and inflammation.

In illustration of the behavior of the conical cervix uteri under labor, two cases were narrated. In one, the cervix and os uteri had returned to their original state, although a fœtus of four-and-a-half or five months' development had been expelled through them. In the other case it was necessary to open the cervix artificially by means of the author's cervical dilator and incisions in order to deliver a full-grown child. In both cases pelvic cellulitis followed labor.

Dr. Baker Brown thanked Dr. Barnes for having brought the subject forward. He agreed with most of what had been stated in the paper; was opposed to dilatation as being inefficient and temporary. He described his mode of operating, which was to place the patient in the lithotomy position, and having introduced a bent speculum, he seized the os with a pair of forceps, and divided it with Simpson's hysterotome. He never divided the internal os; always used a plug of oiled lint to prevent hemorrhage. He regretted that the operation had lately been condemned by a high authority, but believed it was the only efficient and permanent remedy for these painful affections.

Dr. Greenhalgh was surprised to hear the President's opinion that the seat of stricture in these cases was mostly at the external os. He (Dr. Greenhalgh), on the contrary, expressed his conviction that in the great majority of cases the stricture is situated at the internal os, and consequently he recommended division of the internal as well as the external os. After division he usually introduced one of his bilateral expanding stems, which keeps up steady dilatation and prevents contraction. As regarded hemorrhage, which some appeared so to dread, he had never but once met with it, though he had operated in nearly one hundred cases. He always used his own bilateral instrument, which cuts both sides at once. The advantages of his plan of operating were, he believed, extreme exactitude, facility, painlessness, and the avoidance of any personal exposure. He expressed his surprise at the remark of Mr. Brown that he never divided the internal os, when he (Dr. Greenhalgh) had seen him on several occasions freely incise the internal os in the cases under consideration.

Mr. Baker Brown, in answer to Dr. Greenhalgh, said that that gentleman must be mistaken in what he had seen at the London Surgical Home. He repeated that he never in cases of dysmenorrhœa cut through the internal os. Dr. Greenhalgh was evidently confounding this operation with that for fibrous tumor, retroversion, retroflexion, &c., of the uterus, in which he (Mr. Brown) incised freely, and generally through the internal os.

Dr. Routh fully confirmed Dr. Greenlagh's views. For his own part he believed in by far the majority of cases the obstruction was at the *inner* and not the *outer* os; although he did not deny that in some cases of conoid cervix it was present at the external os. He agreed with Dr. Gream in believing that Dr. Sim's plan of operating would occasionally leave a deformed cervix for life; and he did not think it was necessary to cut through the entire cervix. The instrument

Dr. Greenhalgh had invented obviated all danger from hemorrhage. The same was true of his (Dr. Routh's) instrument, which he, however, preferred, because on the bend, and therefore more easy of application in *flexion* cases. A little bleeding was salutary. In most of these cases there was a complication of congestion, which the very incision, by the subsequent hemorrhage, relieved. But there was no doubt that such incisions, however freely made, had a tendency to contract again. Hence it was necessary to keep the cut made patent by some internal uterine pessary, and for some time, it might be for months, so as to allow it to become properly lined with mucous membrane and incontractible. He knew several persons now walking about London with these. In other cases their removal had been followed by conjugal relations and pregnancy, though previously sterile for years. Of the use of sponge-tents and other modes of artificial dilatation, in these cases, he spoke disparagingly. He had seen cellular abscess and death follow their use. They should be used with the greatest caution. He also believed cases of dysmenorrhœa were more common than was generally supposed. Not only was the seat of obstruction more frequently at the *internal* os than the *external*, but, indeed, in many cases, the external os was patent and abnormally so, as shown by Dr. Henry Bennet. And there were many, and by far more numerous, cases of dysmenorrhœa which were in no way due to stricture at either os. As these cases were not, however, referred to by Dr. Barnes, he did not allude to them further. * * * * * * *

Dr. Graily Hewitt thought that the two questions of the treatment of dysmenorrhœa and of sterility by means of incisions of the cervix uteri had been too much mixed up together. He would say a few words first respecting dysmenorrhœa. He believed that in bad cases of dysmenorrhœa the condition present was frequently retention of the fluid in the uterus, and that this retention caused the pain; and he had been at some trouble to prove this. But, on the other hand, he also thought that the condition was capable of being relieved, in most cases, without resort to mechanical treatment of the cervix uteri. The great thing was to diminish the flow of blood, and this could be regulated by general measures; but that there were a few cases in which such general measures were useless he admitted. He differed from the President in reference to the most common seat of the constriction; for although there were cases in which the os uteri was con genitally extremely small and narrow, yet in the larger number of cases of dysmenorrhœa the impediment was situated at the junction of the cervix with the body of the uterus. With regard to the best method of applying mechanical relief when such was required, he thought that cases might be treated on their own merits. Where the cervix was hard and dense, the cutting operation was most indicated, the difficulty being here the greatest; but under other circumstances he preferred the use of tents as dilators. The sea-tangle tent was, he considered, a perfectly safe means of dilating the cervix uteri; but, he would repeat, the cases were few requiring this treatment. As to the mode of incising the cervix or os uteri, here again the operation must be selected according to the case: no one operation would be suited to all circumstances. He would next say a few words on the subject of sterility. It was undoubted that in certain cases the cure of sterility could be effected by dilating the cervix uteri, and much had been said as to the superiority of one mode of dilating over another. The fact was, that so long as the canal of the cervix was a little enlarged, whether by incision or dilatation, the necessary end would be served. The great object was to secure a tolerable patency of the canal at about the menstrual period, when conception was most likely to occur. Supposing the sterility to be cured, the dysmenorrhœa which might be associated with it would be also, in all probability, permanently relieved.

Dr. Marion Sims was surprised at the great difference of opinion expressed by previous speakers as to the seat of the obstruction, but he agreed with those who thought it was at the lower orifice. He then went into some statistical details of his own practice, and laid great stress upon the frequency of curvature of the cervix as a cause of obstruction at the internal os. Though it might, he thought, lead to an actual narrowing of the canal, yet he believed this was an extremely rare occurrence. But in cases of induration and conoidity the os tincæ was abnormally contracted in every case he had seen. Indeed, a conical indurated cervix was incompatible with a normal os tincæ, the existence of the one almost necessarily implying that of the other. With regard to cases referred to by Dr. Gream and Mr. S. Wells, in which the tissue of the cervix had been too largely incised, so that the lips of the os were everted and rolled backwards, he had never seen any such result after his method of operating, but had witnessed it after the metrotome caché; and he attributed it to this—because it cut deeper into the sides of the supra-vaginal portion of the cervix, and so divided the circular muscular fibres, which are naturally antagonistic to the longitudinal fibres. By his (Dr. Sims's) plan of operating, the incisions upward were more superficial, though the opening of the os was about the same in both methods.

The President, in closing the discussion, said that he only directed attention to one class of cases of dysmenorrhœa—that, namely, associated with the peculiar projecting form of cervix uteri, and usually attended by sterility. This was the form that required treatment by incision. The obstruction that required division was the os externum or vaginal portion. The os internum normally was a narrow canal. Dr. Greenhalgh had passed his instrument through it as preliminary to his operation. If it admitted this instrument, the os was of full normal size, and could not require cutting. His (the President's) instrument and operation were perfectly safe and efficient. He thought, after hearing Dr. Sims's remarks, that he had underrated the importance and frequency of flexion at the neck as a cause of obstruction.—*Proceedings of the Obstetrical Society of London*, in *London Lancet.*

